# Editorial: Pancreatic beta-cell dedifferentiation

**DOI:** 10.3389/fendo.2024.1524001

**Published:** 2024-11-29

**Authors:** Xiaoqing Tang, Taiyi Kuo, Zong Wei

**Affiliations:** ^1^ Department of Biological Sciences, Michigan Technological University, Houghton, MI, United States; ^2^ Department of Neurobiology, Physiology, and Behavior, University of California at Davis, Davis, CA, United States; ^3^ Department of Physiology and Biomedical Engineering, Mayo Clinic College of Medicine, Scottsdale, AZ, United States

**Keywords:** type 2 diabetes, type 1 diabetes, beta-cell, dedifferentiation, Ca 2+ signaling, mitochondria

The pathogenesis of both type 1 diabetes (T1D) and type 2 diabetes (T2D) involves a decline in functional β-cell mass, which is essential for insulin secretion and glucose regulation. Historically, apoptosis was considered the primary cause of β-cell loss (1). However, recent evidence highlights a process called β-cell dedifferentiation, in which mature β-cells lose their specialized insulin-secreting identity and revert to a progenitor-like, non-functional state (2). The mechanisms driving this dedifferentiation remain unclear due to the complex interplay between genetic factors and cellular stress. This Research Topic explores key drivers of β-cell dedifferentiation and their role in both T1D and T2D, with a focus on the implications for disease management and potential reversal.

β-cell dedifferentiation is marked by the loss of β-cell identity markers, such as Foxo1, Pdx1, Nkx6.1, and MafA, which are critical for maintaining β-cell function (3, 4). At the same time, there is an upregulation of “disallowed” genes, such as lactate dehydrogenase A (Ldha) and monocarboxylate transporter-1 (Mct1), typically repressed in mature β-cells to prevent inappropriate insulin secretion (5–7). Through dedifferentiation, a subset of terminally differentiated β-cells begin to express lineage precursors such as Neurogenin3 (Ngn3), Oct4, and Nanog, suggesting β-cells regress to a more primitive, less specialized state (2, 8). Furthermore, dedifferentiated beta-cells could begin to express alpha cell signature genes, such as Arx and Gc, and undergo transdifferentiation (9, 10). Environmental factors including chronic inflammation and oxidative stress have been extensively studied in relation to these molecular changes, particularly in animal models and human tissues (3, 11, 12).

In this Research Topic, Patel and Remedi provide a comprehensive review of genetic and cellular stress factors, highlighting the possibility that β-cell dedifferentiation may be reversible. They point out that dedifferentiated cells could potentially redifferentiate into functional β-cells under the right conditions. For example, intensive insulin therapy alone, or in combination with metformin, infusion of human umbilical cord-derived MSCs, as well as ALDH1A3 inhibitors have shown promise in preventing β-cell dedifferentiation and improving glucose tolerance. Moreover, calorie restriction and intermittent fasting have been shown to protect against β-cell dedifferentiation in T2D mouse models, further reinforcing that this process may be reversible.

In addition to dedifferentiation, β-cells have been shown to transdifferentiate into other pancreatic cell types, such as α-, δ-, or pancreatic polypeptide (PP) cells. Human studies of T2D reveal an increased α/β-cell ratio, primarily due to a reduction in β-cell mass. Ex vivo studies suggest that degranulated β-cells can transdifferentiate into α-cells, and this process can be inhibited by knocking down the α-cell marker, Arx. Mouse models also demonstrate that the deletion of certain genes, such as Foxo1, Nkx2.2, Dnmt1, and XBP1, can induce β-to-α or β-to-δ cell transdifferentiation. Conversely, manipulations including PAX4 overexpression or Arx inactivation have been shown to induce α-to-β cell conversion, offering potential avenues for therapeutic intervention. Together, these studies suggest that beta-cell function could be restored by reversing dedifferentiation or transdifferentiation, which opens exciting possibilities for diabetes treatment.

Magnuson and Osipovich discuss that Ca^2+^ signaling is closely linked to metabolic stress-induced β-cell failure. Markers of dedifferentiation, such as Aldh1a3 and Bach2, are upregulated, likely due to chronically elevated intracellular Ca^2+^. Achaete-scute homolog 1 (Ascl1), a Ca^2+^-regulated gene, is activated by Ca^2+^ signaling and contributes to β-cell dysfunction by promoting dedifferentiation while suppressing genes essential for insulin secretion and cell innervation. Notably, removing Ascl1 improved β-cell function under metabolic stress from a high-fat diet, highlighting the importance of maintaining Ca2+ signaling homeostasis for preserving β-cell identity.

Carroll et al. delve into the effects of maternal nutrition on the metabolic health of offspring, particularly in relation to the altered α/β-cell ratio and insulin hypersecretion. They underscore the importance of mitochondrial morphology in β-cell maturation, demonstrating how maternal diet-induced changes at the mitochondrial level can affect β-cell function and influence the offspring’s long-term risk of developing diabetes.

While β-cell dedifferentiation is extensively studied in the context of T2D, its role in T1D remains less understood. T1D is primarily an autoimmune disease in which β-cells are targeted and destroyed by the immune system. Webster and Mirmira analyze existing evidence which suggests that β-cell dedifferentiation may also occur in T1D. Studies using pancreas from T1D donors reveal a significant reduction in insulin-positive cells, yet some residual β-cells persist, even years after disease onset. T1D islets show an increase in non-beta endocrine cells, as well as cells that co-express glucagon and Pdx1, which indicates a potential identity transition between β-cells and α-cells. Additionally, hormone-negative endocrine cells are more prevalent in T1D islets, suggesting that some β-cells may dedifferentiate into other cell types rather than undergoing apoptosis. In T1D, chronic immune attacks may drive β-cells to dedifferentiation as a survival mechanism to evade immune detection, though this comes at the cost of losing insulin secretion and further impairing glucose homeostasis.

In conclusion, β-cell dedifferentiation represents a critical frontier in diabetes research, with the potential to reshape our understanding of both T1D and T2D. The reversibility of this process holds immense promise for developing therapies that preserve or restore β-cell function. In both T2D, where metabolic stress is the main driver of dedifferentiation, and in T1D, where immune evasion plays a role, targeting β-cell dedifferentiation could lead to significant advancements in diabetes treatment and disease management. As research progresses, unraveling the molecular mechanisms underlying β-cell dedifferentiation will be crucial in unlocking new therapeutic strategies aimed at preserving and enhancing β-cell function in diabetic patients.
